# Impact of inflammation, emphysema, and smoking cessation on V/Q in mouse models of lung obstruction

**DOI:** 10.1186/1465-9921-15-42

**Published:** 2014-04-14

**Authors:** Brian N Jobse, Cory AJR McCurry, Mathieu C Morissette, Rod G Rhem, Martin R Stämpfli, Nancy Renée Labiris

**Affiliations:** 1 Department of Medicine, Division of Respirology, McMaster University, Hamilton, Canada; 2 Department of Pathology and Molecular Medicine, McMaster Immunology Research Centre, McMaster University, Hamilton, Canada; 3 Firestone Institute for Respiratory Health at St. Joseph’s Healthcare, Hamilton, Canada

**Keywords:** Chronic obstructive pulmonary disease, Gas exchange, Lung function, Perfusion, Single photon emission computed tomography, Ventilation

## Abstract

**Background:**

Chronic obstructive pulmonary disease (COPD) is known to greatly affect ventilation (V) and perfusion (Q) of the lung through pathologies such as inflammation and emphysema. However, there is little direct evidence regarding how these pathologies contribute to the V/Q mismatch observed in COPD and models thereof. Also, little is known regarding how smoking cessation affects V/Q relationships after inflammation and airspace enlargement have become established. To this end, we have quantified V/Q on a per-voxel basis using single photon emission computed tomography (SPECT) in mouse models of COPD and lung obstruction.

**Methods:**

Three distinct murine models were used to investigate the impact of different pathologies on V/Q, as measured by SPECT. Lipopolysaccharide (LPS) was used to produce neutrophilic inflammation, porcine pancreatic elastase (PPE) was used to produce emphysema, and long-term cigarette smoke (CS) exposure and cessation were used to investigate the combination of these pathologies.

**Results:**

CS exposure resulted in an increase in mononuclear cells and neutrophils, an increase in airspace enlargement, and an increase in V/Q mismatching. The inflammation produced by LPS was more robust and predominantly neutrophilic, compared to that of cigarette smoke; nevertheless, inflammation alone caused V/Q mismatching similar to that seen with long-term CS exposure. The emphysematous lesions caused by PPE administration were also capable of causing V/Q mismatch in the absence of inflammation. Following CS cessation, inflammatory cell levels returned to those of controls and, similarly, V/Q measures returned to normal despite evidence of persistent mild airspace enlargement.

**Conclusions:**

Both robust inflammation and extensive airspace enlargement, on their own, were capable of producing V/Q mismatch. As CS cessation resulted in a return of V/Q mismatching and inflammatory cell counts to control levels, lung inflammation is likely a major contributor to V/Q mismatch observed in the cigarette smoke exposure model as well as in COPD patients. This return of V/Q mismatching to control values also took place in the presence of mild airspace enlargement, indicating that emphysematous lesions must be of a larger volume before affecting the lung significantly. Early smoking cessation is therefore critical before emphysema has an irreversible impact on gas exchange.

## Introduction

Ventilation (V) and perfusion (Q) are fundamental physiological processes within the lung contributing to gas exchange, and the relationship between these processes is dysfunctional in patients with chronic obstructive pulmonary disease (COPD) [1]. Cigarette smoke (CS) is a primary risk factor for the disease, prolonged exposure to which can lead to airway inflammation, airspace enlargement, and several other pathologies [2], ultimately resulting in irreversible airflow limitation [3,4]. Cessation of cigarette smoking is capable of slowing the progression of COPD but years of cessation are often necessary before improvements to airflow limitation, inflammatory state, infection risk, and cardiovascular comorbidities are seen [5,6]. To better understand the benefits and limitations of smoking cessation, the impact on V/Q requires investigation in the context of the pathologies associated with cigarette smoke exposure.

V/Q relationships in the lung can be measured by several non-invasive methods but these techniques are not commonly used in clinical practice, aside from the diagnosis of pulmonary embolism; quantification of results, even in clinical research, is rare. Rodríguez-Roisin *et al.*[7] made use of the multiple inert gas elimination technique (MIGET) to quantitatively demonstrate that V/Q is a sensitive measure of the earliest stages of COPD. A more widely available methodology for V/Q can be performed in nuclear medicine departments utilising single photon emission computed tomography (SPECT) to provide three-dimensional maps of ventilation and perfusion [8,9]. Jogi *et al.*[10] have shown the ability of this technique to identify early disease and stage disease severity in COPD patients. Further, Suga *et al.*[11] have quantified the impact of emphysema on V/Q in the lungs of COPD patients.

Modelling aspects of COPD in mice provides the means by which to investigate the individual pathologies that make up this heterogeneous disease [12]. Using a methodology adapted from clinical V/Q SPECT to a murine model, our laboratory has confirmed the utility of this technique in measuring changes in V/Q with age [13] and in the context of prolonged cigarette smoke exposure [14].

Understanding the mechanisms behind V/Q mismatch in COPD-associated pathologies is an important step in furthering the ability to diagnose and treat this widespread and burdensome disease. In the current study, the impact of smoking cessation on V/Q mismatching has been investigated. In addition, the contributions of inflammation and airspace enlargement have been examined, by employing simple models for these pathologies, to provide insight into the dysfunction associated with cigarette smoke exposure. We have sought to explore the V/Q relationships associated with cigarette smoke exposure using these models alongside cellular and structural assessments, using both traditional and non-invasive methods.

## Materials and methods

### Animals

Specific pathogen-free 10–12 week old female BALB/c mice were purchased from Charles River Laboratories (Senneville, QC, Canada). The studies were approved by McMaster University’s Animal Research Ethics Board in accordance with the Canadian Council on Animal Care guidelines.

### Cigarette smoke exposure protocol

Mice were exposed to cigarette smoke 5 days/week using a SIU48 whole body exposure system (Promech Lab, Vintrie, Sweden). Details of the exposure protocol have been reported previously [15]. Control animals were exposed to room air only. Following 24 weeks of smoke exposure, mice were divided into two groups, continued smoke exposure and smoke cessation, and studied for 16 weeks. Controls continued to receive room air.

### Lipopolysaccharide exposure protocol

To model neutrophilic lung inflammation, mice intranasally received either 10.5 μg of LPS (Sigma-Aldrich, Oakville, ON, Canada) in 35 μL of sterile phosphate-buffered saline (PBS) or PBS only. Animals were imaged 24 hours post LPS exposure and sacrificed immediately after imaging.

### Porcine pancreatic elastase exposure protocol

To model emphysema, mice intranasally received 4 units of PPE (EPC Inc., Owensville, MO, USA) in 30 μL of sterile PBS or PBS only. After exposure mice were left for a period of 45 days prior to acquisition of data.

### Imaging protocol and per-voxel image analysis

Imaging was performed as previously described [13] with minor modifications. SPECT scans were acquired on an X-SPECT system (Gamma Medica, Northridge, CA, USA). Technegas™ and ^99m^Tc-macroaggregated albumin were used to provide the distributions of V and Q, respectively. CT images were acquired for both SPECT scans, also on the X-SPECT. SPECT and CT images were reconstructed, fused, and co-registered as previously described [13]. A ‘Lung’ region of interest (ROI) was produced for the ventilation CT images using Amira 5.1 software (Visage Imaging, Andover, MA, USA) and used during co-registration and analysis. V/Q ratios were calculated using normalised V and Q frequencies. To assess emphysema in CT images, volumes of low attenuation (VLA) were calculated by summing the percentage of lung volume less than -400HU. Additional details regarding this threshold and other specifics of the methods used are provided in the Additional file 1.

### Collection and measurement of specimens

Mice were sacrificed at the experimental endpoints indicated in the results. Bronchoalveolar lavage (BAL) was collected and measures of airspace enlargement were made (Pneumometrics software V.1, Hamilton, Ontario, Canada) from haemotoxylin and eosin (H&E) stained lung histology sections as described previously [14]. BAL cell counts and the histological data for animals at 24 weeks CS have been reported previously [14]. Additional detail regarding lung fixation, histological assessment, and BAL quantification is provided in the Additional file 1.

### Data analysis

Data were expressed as the mean ± SEM. Statistical significance was determined by an unpaired, two-tailed t-test in Prism (Graphpad Software Inc, La Jolla, CA, USA) when comparing age-matched experimental groups. For cessation-related data, a one-way ANOVA with Tukey post-hoc test was performed. p < 0.05 was considered statistically significant for all statistical tests. The number of mice studied is described in Table one, found in the Additional file 1.

## Results

### Cigarette smoke exposure caused V/Q mismatch, inflammation, and airspace enlargement

**Figure 1:**
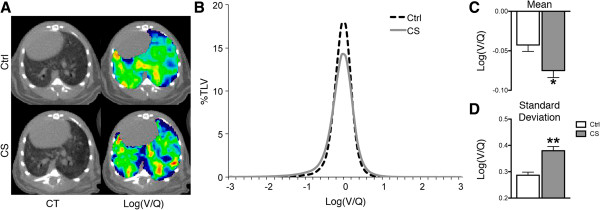
**Impact of 24 weeks smoke exposure on lung function. A** Representative axial CT (left) and log(V/Q) (right) images for control (top) and cigarette smoke exposed (bottom) animals after 24 weeks. Green represents matched V/Q while colours progressing towards purple and red represent increasing values of low and high V/Q, respectively. **B** Log(V/Q) distributions for animals at 24 weeks. **C** Average mean log(V/Q) values. **D** Average log(V/Q) standard deviation values. *p < 0.05, **p < 0.01 by two-tailed t-test compared to age-matched controls.

**Figure 2:**
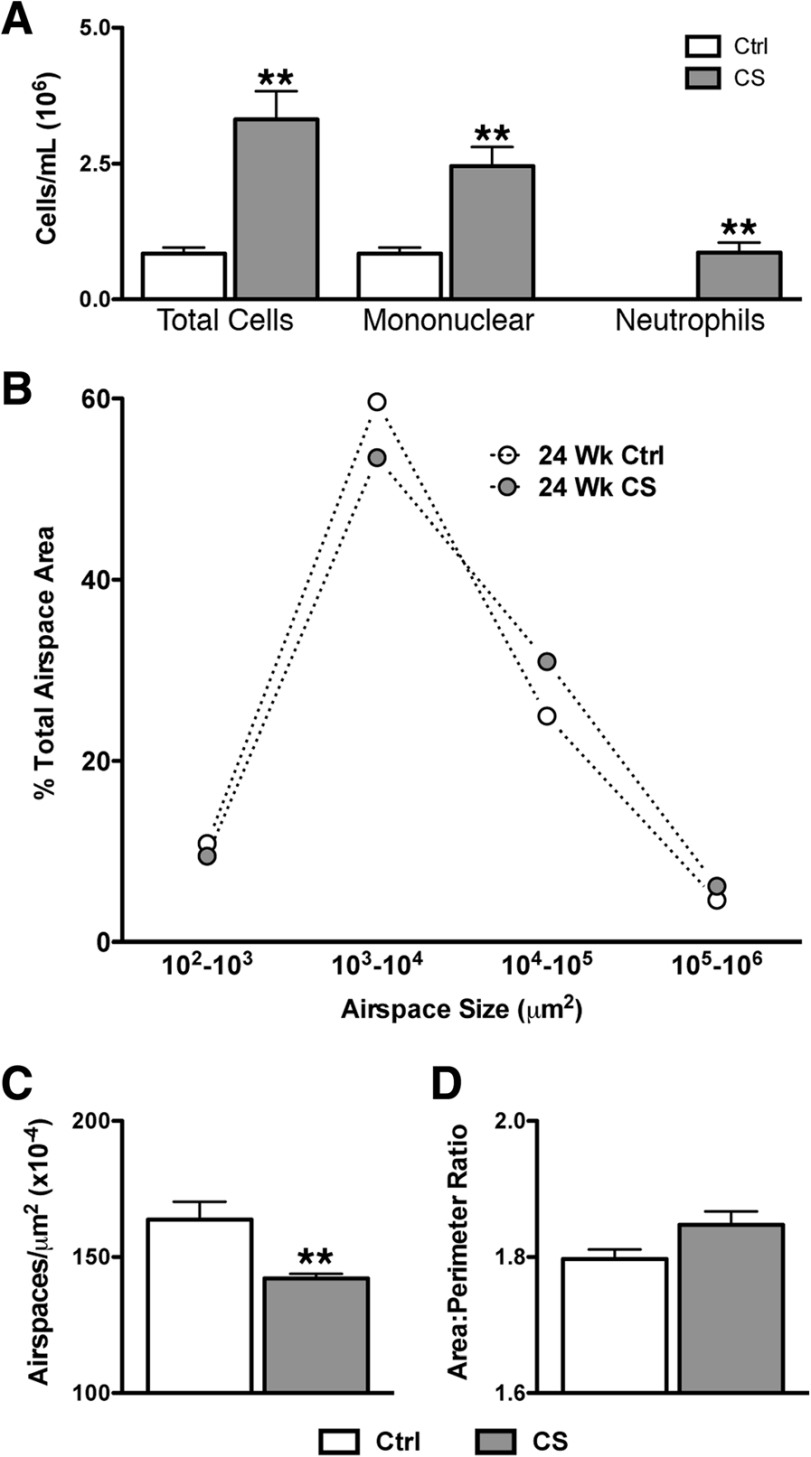
**Inflammation and airspace enlargement after 24 weeks smoke exposure. A** BAL total cells, mononuclear cells, and neutrophils at 24 weeks. **B** Average airspace area distributions by logarithmic bins of airspace size for 24 weeks, describing whole slice histology. **C** Average airspaces per unit area. **D** Average area to perimeter ratio produced from analysis of whole slice histology at 24. **p < 0.01 by two-tailed t-test compared to age-matched controls.

Mice were exposed to CS for 24 weeks to establish the degree of V/Q mismatching and the lung conditions in which mismatching takes place. Exposure to cigarette smoke for a period of 24 weeks elicited significant V/Q mismatching but no density changes were observed in CT images compared to controls (Figure 1A). Quantification of log(V/Q) curves (Figure 1B) yielded a significant decrease in the mean and a significant increase in the standard deviation of the data (Figure 1C&D). Analysis of BAL fluid demonstrated that 24 weeks of smoke exposure caused robust inflammation, with a total cell count of 3.3 ± 0.5 ×10^6^ compared to 0.8 ± 0.1 ×10^6^ for controls. Similarly, increases were observed for both mononuclear cells and neutrophils (Figure 2A). A shift towards greater airspace size was observed histologically in the distribution of airspace area (Figure 2B) and statistically confirmed by quantification of the airspace area beyond the control 75^th^ percentile (data not shown). In addition, a decrease of the number of airspaces per unit area (Figure 2C) was seen. Thus, CS exposure caused V/Q mismatch in the context of both inflammation and a small but significant degree of airspace enlargement.

### Inflammation alone caused V/Q mismatch

**Figure 3:**
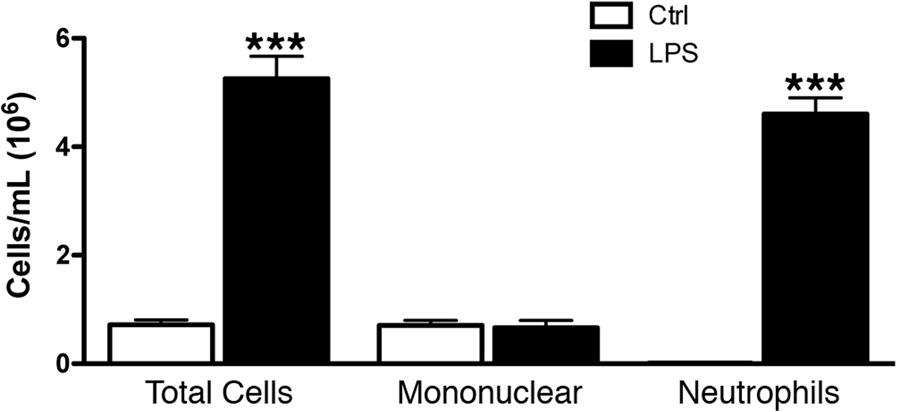
**BAL cell counts for LPS exposure.** Total cells and the associated number of mononuclear cells and neutrophils are shown. ***p < 0.001 by two-tailed t-test compared to age-matched controls.

**Figure 4:**
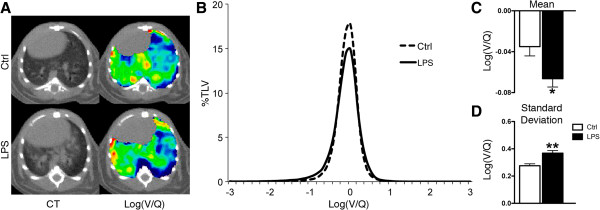
**Functional impact of LPS exposure. A** Representative axial CT (left) and log(V/Q) (right) images for control (top) and LPS exposed animals (bottom). Green represents matched V/Q while colours progressing towards purple and red represent increasing values of low and high V/Q, respectively. **B** Log(V/Q) distributions. **C** Average mean log(V/Q) values. **D** Average log(V/Q) standard deviation. *p < 0.05, **p < 0.01 by two-tailed t-test compared to age-matched controls.

The effect of inflammation on V/Q mismatch was investigated by exposing mice to LPS. LPS exposure produced a total cell count of 5.3 ± 0.4 ×10^6^ compared to 0.7 ± 0.1×10^6^ for control animals; this increase was almost entirely neutrophilic (Figure 3). The increased inflammation associated with LPS was also apparent in CT images, as depicted by peribronchial increases in density (Figure 4A). The log(V/Q) distribution demonstrated that this inflammation was capable of altering lung function (Figure 4B) and significant changes were observed in both the mean and standard deviation of these data (Figure 4C and D). Thus, neutrophilic inflammation alone was capable of causing V/Q mismatch.

### Airspace enlargement alone caused V/Q mismatch

**Figure 5:**
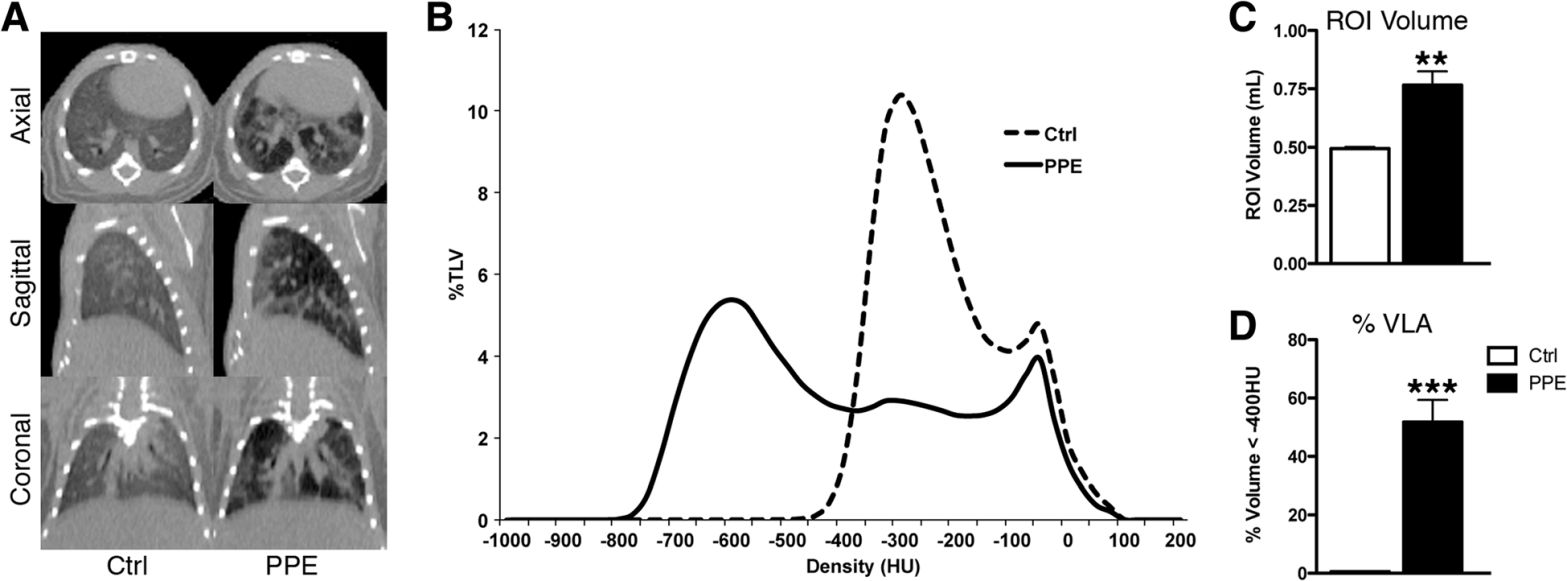
**Density-based analysis of PPE imaging data. A** Representative axial, sagittal, and coronal CT slices from PPE-exposed and age-matched controls. **B** Average Hounsfield unit (HU) density distributions for control and PPE-exposed groups. **C** Volume of the lung region of interest (ROI). **D** Quantification of percentage of volume with density values less than -400HU, A.K.A. percentage volume of low attenuation (%VLA), signifying severe airspace enlargement. **p < 0.01, ***p < 0.001 by two-tailed t-test compared to age-matched controls.

The role of airspace enlargement in V/Q mismatching was next investigated as a major pathology associated with COPD. To produce airspace enlargement greater than the levels observed after exposure to cigarette smoke, mice were exposed to PPE. Emphysema-like lesions were readily apparent in the CT images (Figure 5A). While the control group was described by a bimodal distribution representing air- and tissue-filled regions within the lung segmentation, the PPE group showed a large leftward shift towards lower density values, indicating a greater extent of air within the lung (Figure 5B). The lung ROI volume analysed was significantly greater in PPE-exposed animals than in controls (Figure 5C). When the percentage of volume below -400HU was calculated, a threshold describing emphysematous lesions, a significant increase was found in PPE animals (Figure 5D) with 21.9 ± 2.4% of the lung volume below -400HU compared to 0.6 ± 0.1% in controls.

**Figure 6:**
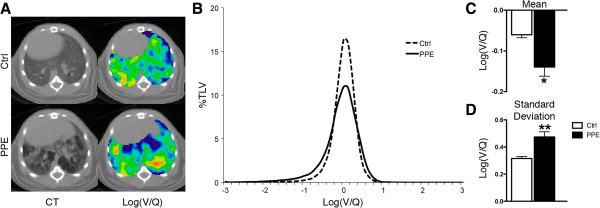
**PPE-induced damage and V/Q mismatch. A** Representative axial CT (left) and log(V/Q) (right) images for control (top) and PPE exposed (bottom) animals. Green represents matched V/Q while colours progressing towards purple and red represent increasing values of low and high V/Q, respectively. **B** Log(V/Q) distributions for animals 45 days after PPE exposure. **C** Average mean log(V/Q) values. **D** Average log(V/Q) standard deviation values following PPE exposure. *p < 0.05, **p < 0.01, by two-tailed t-test compared to age-matched controls.

Log(V/Q) measurements were affected by the altered lung structure produced by PPE exposure, though no consistent pattern was observed relating emphysematous lesions to mismatched V/Q (Figure 6A). A broadening of the log(V/Q) distribution was seen (Figure 6B) and was further described by a significant decrease in the mean and an increase in the standard deviation (Figure 6C and D). No difference was observed in BAL inflammatory levels between control and PPE exposed mice at the experimental endpoint (data not shown). Therefore, emphysematous lesions were capable of producing V/Q mismatch, which appeared to be distributed throughout the lung.

### Cigarette smoke cessation resolved V/Q mismatch

**Figure 7:**
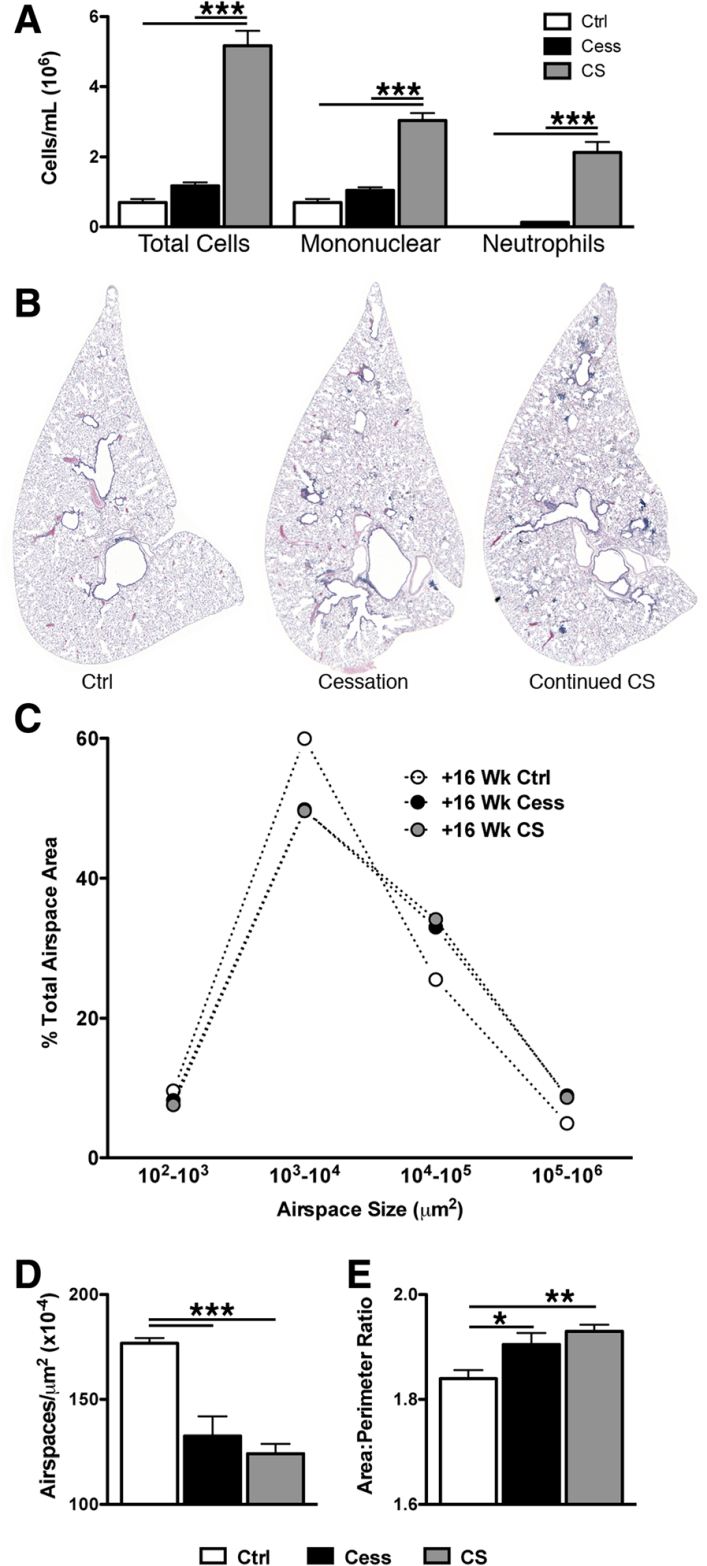
**Inflammation and airspace enlargement after 16 weeks cessation. A** BAL total cells, mononuclear cells, and neutrophils at 16 weeks post 24 week smoke exposure. ***p < 0.001 by one-way ANOVA with Tukey post-hoc. **B** Representative, H&E stained, whole slice histology from a control (left), cessation (middle), and continuing smokers at the +16 week time-point. **C** Average airspace area distributions by logarithmic bins of airspace, describing whole slice histology. **D** Average airspaces per unit area and **E** average area to perimeter ratio produced from analysis of whole slice histology. *p < 0.05, **p < 0.01, ***p < 0.001 by one-way ANOVA with Tukey post-hoc.

Cessation of cigarette smoke exposure was next examined to determine the relative roles of inflammation and airspace enlargement to the V/Q mismatching observed after 24 weeks of exposure. Following 16 weeks of cessation, significant decreases were observed in the BAL total cell count, as well as within the mononuclear and neutrophil compartments, bringing these levels back to those observed in controls (Figure 7A). However, the lungs of smoking cessation animals still showed evidence of bronchial associated lymphoid tissue (Figure 7B); these immune structures were present after 24 weeks of smoke exposure and did not resolve over this period of cessation. Histological analysis of airspace enlargement demonstrated that continuing smoke and cessation groups had similar airspace area distribution profiles (Figure 7C). The percentage of area above the control 75^th^ percentile was significantly increased in both of these groups compared to controls (data not shown). Similarly, the decrease in the number of airspaces per unit area (Figure 7D), and an increase in the area to perimeter ratio (Figure 7E), confirmed that the airspace enlargement seen at 24 weeks was still apparent after 16 weeks of cessation.

**Figure 8:**
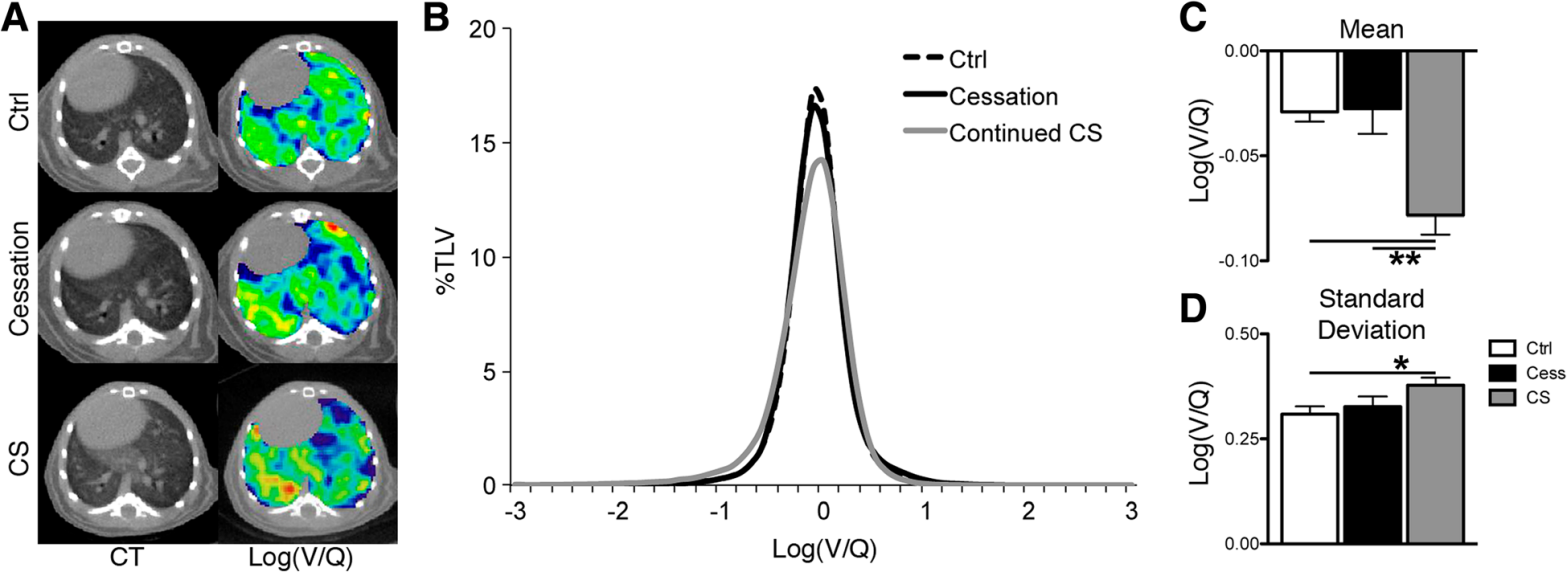
**Impact of 16 weeks cessation on lung function. A** Representative axial CT (left) and log(V/Q) (right) images for control (top), cessation (middle), and continuing cigarette smoke exposed (bottom) animals after +16 weeks. Green represents matched V/Q while colours progressing towards purple and red represent increasing values of low and high V/Q, respectively. **B** Log(V/Q) distributions for animals at +16 weeks. **C** Average mean log(V/Q) values. **D** Average log(V/Q) standard deviation values. *p < 0.05, **p < 0.01 by one-way ANOVA with Tukey post-hoc.

There was no discernible difference in log(V/Q) distributions between control animals and those that stopped smoking after 24 weeks of cigarette smoke exposure while continuing smokers maintained the altered log(V/Q) distribution (Figure 8A&B). With smoking cessation a significant increase of the mean log(V/Q) value, from −0.08 ± 0.01 after 24 weeks smoke exposure to −0.03 ± 0.01 after 16 weeks of smoking cessation, and a significant decrease of the standard deviation of this data, from 0.38 ± 0.02 to 0.33 ± 0.02 after cessation, was observed. Thus, cessation for 16 weeks returned V/Q mismatching back to control values.

## Discussion

Ventilation and perfusion of the lung can be compromised in COPD, and the capability of matching these processes dysregulated. The objective of the current study was to investigate the V/Q perturbations associated with two of the major pathologies associated with COPD using mouse models of neutrophilic inflammation and emphysema. Further, the impact of smoking cessation was described and evidence gathered regarding the relative roles of inflammation and airspace enlargement in V/Q mismatching.

The mouse models employed in the studies presented were relatively simple in nature to allow for effective interpretation of results. These models, utilising LPS, PPE, and cigarette smoke, are all well-established and the impact of these exposures on resistance to airflow, the immune system, and other aspects of the lung have been reviewed previously [12]. Investigation of V/Q relationships in these models adds to the understanding of the consequences of pathological disruption on the potential for gas exchange. The LPS and PPE models, causing inflammation and airspace enlargement, respectively, were used as examples of severe pathology, so the V/Q mismatching observed was not surprising. It is important to note that the extent and distribution of pathology associated with these administered reagents does not necessarily reflect the pathologies found in COPD, but demonstrate that each causes V/Q mismatching. Cigarette smoke, on the other hand, caused less pronounced inflammation and only subtle airspace enlargement but nevertheless caused V/Q mismatching similar to that observed in the other models employed.

A seminal article by Wright and Sun [16] investigated long-term smoking cessation in guinea pigs and found that airspace enlargement persists in ex-smokers while pulmonary function increases over their smoking counterparts. Similarly, we found that smoking cessation resulted in a return to normal lung function, as measured by V/Q relationships, and decreased inflammation. However, it has been established that other pathological markers, such as bronchial-associated lymphoid tissue, remain after smoking cessation [17]. Likewise, the airspace enlargement present after 24 weeks of cigarette smoke exposure persisted after smoking cessation, but these emphysematous lesions caused by cigarette smoke exposure were mild compared to elastase induced airspace enlargement and likely were not sufficient enough to contribute to V/Q mismatch. It is possible that the resolution of the imaging methodology was unable to detect the mismatch caused by these small, persistent structural changes; however, if airspace enlargement were to continue, V/Q mismatch and impaired gas exchange would eventually ensue, indicating that smoking cessation in human patients is critical before emphysematous lesions are present; at these early stages of disease the V/Q mismatch from inflammation could resolve leaving the gas exchange capabilities of the lung largely intact.

Work by Suga *et al.*[11] has begun to explore the V/Q relationships in COPD patients with advanced emphysema but our results suggest that changes in V/Q may not be apparent, due to airspace enlargement alone, until this pathology has progressed substantially as evidenced by the lack of V/Q mismatching in the presence of mild emphysema in cessation mice. Also of interest, the relationship between volumes of low x-ray attenuation and V/Q was not apparent in the comparison of log(V/Q) images to CT images in PPE-exposed animals. It is possible that regions neighbouring emphysematous volumes are unable to function properly, leading to the V/Q mismatch observed. This warrants further investigation and V/Q SPECT/CT provides the necessary tools to address this concern. It is now understood that emphysema progression continues after smoking cessation [18,19], so development of methods that can be used clinically to track and understand this pathology are paramount.

The impact of inflammation on V/Q status is also an important topic that is not yet well understood. While LPS caused a greater inflammatory reaction than cigarette smoke, as observed in both BAL measurements and CT images, it did not elicit a V/Q disturbance greater than that of cigarette smoke. It is likely that the distribution of this inflammation is an important factor, especially as it pertains to the small airways; constriction of the small airways is undoubtedly heterogeneous and would lead to heterogeneous ventilation patterns. As airflow resistance is inversely proportionate to the radius of the airway to the fourth power, as described by Poiseuille’s equation, even slight changes in the lumen of small airways could alter the distribution of ventilation and impact V/Q relationships. Investigations by Gaschler *et al.*[20] demonstrated that mucus secretion is not present within the small airways after 8 weeks of cigarette smoke exposure, but there is thickening of the epithelial layer [21]. V/Q mismatching is present after 8 weeks smoke exposure in this model [14], but further investigation into the mechanisms by which inflammation could affect airflow in this manner is required. While LPS-derived inflammation caused V/Q mismatch, likely through airflow obstruction, it is important to note that cigarette smoke contains additional components, such as nitric oxide, that could interfere with vascular mechanisms, such as hypoxic vasoconstriction, leading to inadequate matching of perfusion to ventilation [22,23]. Thus, the V/Q mismatch observed in this model of cigarette smoke exposure is likely dependent on both inflammation and an alteration in perfusion, though greater investigation is still necessary.

In comparison to clinical findings, our data are consistent with those previously reported by Rodríguez-Roisin *et al.*[7] using MIGET where V/Q was shown to be sensitive to GOLD stage I and that mismatching increased with GOLD staging severity. The authors also provided evidence that the V/Q abnormalities seen in GOLD stage I were associated with smaller airways, alveolar airspaces, and blood vessels. Our data suggests that inflammation could play a large role in the V/Q mismatching observed in early COPD, while other pathologies, such as emphysema and small airway fibrosis, become a principal cause of V/Q mismatching in the later stages of COPD. SPECT V/Q has previously been shown, through work by Petersson *et al.*[24], to closely parallel MIGET results such as those described above but it is important to note that our protocol did not contain a measurement of total cardiac output. As such, this preclinical technique approximates overall V/Q distribution. Nevertheless, due to the consistent attributes inherent in an experimental model, as compared to human subjects, we believe that our V/Q results are representative of the state of the lungs in the contexts described.

The pulmonary processes of ventilation and perfusion are both affected by long-term exposure to cigarette smoke. Cessation of cigarette smoking results in a return of V/Q assessed lung function to normal, but the pathological consequences of continued exposure eventually lead to structural damage and functional impairment. It is possible that these pathologies could be detected early with the aid of V/Q methods and cessation initiated before major damage permanently alters pulmonary lung function.

## Conclusions

While models of COPD cannot reproduce the disease itself in entirety, they allow research to address both the pathogenesis of the disease and the constituent pathologies therein. We have demonstrated that both inflammation and emphysematous lesions can contribute to V/Q mismatching and that cigarette-smoking cessation prior to large-scale structural changes can reverse the V/Q imbalance. Spatial methodologies utilising V/Q, especially those coupled to anatomical imaging methods, can provide information not accessible by other means. The ability to translate knowledge of lung structure and function garnered in preclinical models to that seen in clinical disease could provide better diagnosis, treatment, and understanding of chronic respiratory diseases such as COPD.

## Abbreviations

99mTc: 99-metastable technetium; ANOVA: Analysis of variance; BAL: Broncho-alveolar lavage; COPD: Chronic obstructive pulmonary disease; CS: Cigarette smoke; CT: Computed tomography; Log: Base 10 logarithm; LPS: Lipopolysaccharide; MAA: Macroaggregated albumin; MIGET: Multiple inert gas elimination technique; PBS: Phosphate buffered saline; PPE: Porcine pancreatic elastase; SEM: Standard error of the mean; SPECT: Single photon emission computed tomography; VLA: Volume of low attenuation; V/Q: Ventilation/Perfusion.

## Competing interests

Financial Support: Firestone Institute of Respiratory Health – AstraZeneca Collaboration Unrestricted Grant; The Canadian Institutes of Health Research; N.R. Labiris holds an internal Department of Medicine Career Award.

## Authors’ contributions

BNJ was involved in concept and design, experimentation and collection of biological and imaging data, analysis, drafting and review of the manuscript. CAJRM was involved in experimentation and collection of biological and imaging data. MCM was involved in experimentation and collection of biological data as well as review of the manuscript. RGR was involved in collection and analysis of imaging data. MRS contributed to concept and design and review of the manuscript. NRL contributed to concept and design and review/editing of the manuscript. All authors read and approved the final manuscript.

## Supplementary Material

Additional file 1 An expanded Materials and Methods section is available for additional information regarding many of the models and techniques employed in this study. Click here for file
