# Supplementary material for: Impact of inflammation, emphysema, and smoking cessation on V/Q in mouse models of lung obstruction
Source: Respir Res. 2014 Apr 14;15(1):42. doi: 10.1186/1465-9921-15-42 (PMC4021179; doi:10.1186/1465-9921-15-42)
Supplement: Additional file 1 — An expanded Materials and Methods section is available for additional information regarding many of the models and techniques employed in this study. [file 1465-9921-15-42-S1.doc]

**Impact of inflammation, emphysema, and smoking cessation on V/Q**

**in mouse models of lung obstruction**

Brian N. Jobse, Cory A.J.R. McCurry, Mathieu C. Morrisette,

Rod G. Rhem, Martin R. Stämpfli, and N. Renée Labiris.

**Online Supplement**

**MATERIALS AND METHODS (EXPANDED):**

**Animals*:*** Specific pathogen-free 10-12 week old female BALB/c mice were purchased from Charles River Laboratories (Senneville, QC, Canada). Mice were acclimatized to housing conditions for a period of 2 weeks prior to experimentation. Housing conditions consisted of a 12:12 hour light:dark cycle and unrestricted food and water. The studies described were approved by the Animal Research Ethics Board of McMaster University (Hamilton, ON, Canada) in accordance with the guidelines of the Canadian Council on Animal Care.

**Cigarette smoke exposure protocol:** Mice were exposed to the smoke generated from 12 2R4F reference cigarettes (University of Kentucky, Lexington, KY, USA), with the filters removed, for 50 minutes twice daily, 5 days/week using a SIU48 whole body exposure system (Promech Lab, Vintrie, Sweden). Details of the exposure protocol have been reported previously . Control animals were exposed to room air only. Following 24 weeks of smoke exposure, mice were divided into two groups: one subset continued smoke exposure, while another ceased smoke exposure and was exposed to room air only. Controls continued to receive room air. This treatment continued for 16 weeks beyond the original 24 week exposure for a total protocol length of 40 weeks. Average total particulate matter during exposure was 653μg/L. Smoke exposure in this system is well tolerated and results in increased levels of carboxyhaemoglobin and cotinine .

**Lipopolysaccharide exposure protocol:** To model neutrophilic lung inflammation, naïve 26 week old mice were anaesthetized with isoflurane (Baxter, Mississauga, ON, Canada) and 10.5μg of LPS (Sigma-Aldrich, Oakville, ON, Canada) in 35μL of sterile phosphate-buffered saline (PBS) was administered intranasally. Controls received 35μL PBS alone. Animals were imaged 24 hours post LPS exposure and sacrifice occurred immediately after imaging was performed.

**Porcine pancreatic elastase exposure protocol:** Mice were anaesthetized with isoflurane and 4 units of PPE (EPC Inc., Owensville, MO, USA) in 30μL of sterile PBS was administered intranasally. Controls received 30μL PBS alone. After exposure mice were left for a period of 45 days prior to acquisition of data.

**Imaging protocol:** Imaging was performed as previously described with minor modifications. Ketamine/Xylazine (90mg/kg, 6mg/kg) anaesthetized mice freely breathed Technegas^TM^ (Cyclomedica, Lucas Heights, NSW, Australia) (0.04-0.12MBq/mL), from a nose cone within a sealed and HEPA filtered (PALL corp., Missisauga, ON, Canada) acrylic chamber for a period of 15 minutes. Prior to ventilation, oxygen was mixed into the Technegas^TM^ to a level of approximately 20% O_2_. A Rodent Ventilator (Model 683, Harvard Apparatus, Holliston, MA, USA) provided flow to the nose cone (0.02L/min, 125 strokes/min). SPECT scans were acquired on an X-SPECT system (Gamma Medica, Northridge, CA, USA) using pinhole collimators and a radius of rotation of 3.5cm. A high quality ventilation CT was collected immediately after the SPECT scan, also acquired on the X-SPECT system with x-ray tube characteristics of 75kVp and 220μA. Following ventilation SPECT and CT scans, mice were injected with 11-15MBq of ^99m^Tc-MAA via the tail vein. Perfusion imaging entailed a CT and SPECT scan. Supplemental gaseous anaesthesia was used (isoflurane 1%, 1L/min) to ensure sedation throughout the imaging procedure.

SPECT and CT images were reconstructed, fused, and co-registered as previously described . Calibration of each CT image for Hounsfield Unit (HU) scaling was performed using a water-filled tube included in each scan and air within the field of view but external to the animal. A ‘Lung’ region of interest (ROI) was produced for the ventilation CT images, as described previously, using Amira 5.1 software (Visage Imaging, Andover, MA, USA). Fusion parameters between SPECT and CT were found for an experimental time-point by use of a phantom of ^99m^Tc-pertechnetate in capillary tubes at varying angles to each other. CT and SPECT images were acquired for phantoms, reconstructed as described, and fused by in-house, mutual information (MI) maximizing software. These parameters were then used for both ventilation and perfusion data sets. The V and Q data sets were then co-registered by repeating the MI maximization process through rigid body transformation of their respective CT images within the lung ROI.

**Quantitative Per-Voxel Image Data Analysis:** V and Q values were converted to relative frequencies by dividing the activity value of each voxel by the total activity in the lung ROI and then multiplying by 100. V/Q ratios were calculated using the relative frequency of both V and Q and a base 10 logarithm was applied to provide a log-normal distribution. Analysis of the log(V/Q) data involved calculation of the mean and standard deviation of the distribution. Because of the use of the logarithmic scale any voxel where only V equalled zero was set to a log(V/Q) ratio of -∞ and any voxel where only Q equalled zero was set to a log(V/Q) ratio of +∞; these values were not included in the calculation of the log(V/Q) mean and standard deviation. Any voxels where both V and Q equalled zero were noted and given a log(V/Q) value of zero; this value was negligible across all models (<0.05%). To assess emphysema in CT images, volumes of low attenuation (VLA) were calculated by summing the percentage of lung volume less than -400HU, as control animals for the PPE study had less than 1% of volume associated with values beyond this Hounsfield unit threshold.

**Collection and measurement of specimens:** Mice were sacrificed at the experimental endpoints indicated in the results section and bronchoalveolar lavage (BAL) was collected by a method detailed previously . Briefly, the lungs were removed and lavaged twice with PBS (0.45mL total). Total cell counts were determined by haemocytometer and differential cell counts were determined after cytocentrifugation and staining from at least 500 leukocytes using standard haemocytological procedures to classify the cells as neutrophils, eosinophils, or mononuclear cells.

Measures of airspace enlargement, including airspace area distributions, airspaces per unit area, and the mean area to perimeter ratio, were made by use of Pneumometrics software (Pneumometrics© software V.1, Hamilton, Ontario, Canada) from haemotoxylin and eosin (H&E) stained lung histology sections, also by a method described previously . Briefly, the lungs were inflated with formalin under a constant pressure and the left lobe segmented and cross sectioned at the same points of reference for each animal. Each result for an animal therefore represents the average of 4 histological slices obtained for the left lung lobe with major airways and blood vessels identified by the user and removed within the software. The distribution of airspace area was analyzed by determining the average 75^th^ percentile of control animals at 24 weeks (~15000 μm^2^); this threshold was used to calculate the total percentage of area beyond the control 75^th^ percentile for all groups. Bronchoalveolar lavage (BAL) cell counts and the histological airspace area distributions for animals at 24 weeks have been reported previously .

**Data Analysis:** Data were expressed as the mean±SEM. Statistical significance was determined by an unpaired, two-tailed t-test in Prism (Graphpad Software Inc, La Jolla, CA, USA) when comparing age-matched experimental groups. For cessation-related data, a one-way ANOVA with Tukey post-hoc test was performed. p<0.05 was considered statistically significant for all statistical tests. The number of mice studied is described in Table 1.

**REFERENCES:**

1. Botelho FM, Gaschler GJ, Kianpour S, Zavitz CC, Trimble NJ, et al. (2010) Innate immune processes are sufficient for driving cigarette smoke-induced inflammation in mice. Am J Respir Cell Mol Biol 42: 394-403.

2. Jobse BN, Rhem RG, McCurry CA, Wang IQ, Labiris NR (2012) Imaging Lung Function in Mice Using SPECT/CT and Per-Voxel Analysis. PLoS One 7: e42187.

3. Jobse BN, Rhem RG, Wang IQ, Counter WB, Stampfli MR, et al. (2013) Detection of lung dysfunction using ventilation and perfusion SPECT in a mouse model of chronic cigarette smoke exposure. J Nucl Med 54: 616-623.

| Experiment | Group | V/Q & CT | BAL | Histology |
| --- | --- | --- | --- | --- |
| 24 weeks smoke exposure | Control | 5 | 4 | 4 |
|  | Smoke | 12 | 7 | 6 |
| 40 weeks smoke exposure | Control | 4 | 7 | 7 |
|  | Cessation | 4 | 4 | 4 |
|  | Smoke | 7 | 7 | 7 |
| LPS exposure | Control | 4 | 5 | n/a |
|  | LPS | 5 | 4 | n/a |
| PPE exposure | Control | 4 | n/a | n/a |
|  | PPE | 4 | n/a | n/a |

**Table 1: Subject numbers used in experimentation**
